# Regulation of root development in nitrogen-susceptible and nitrogen-tolerant sweet potato cultivars under different nitrogen and soil moisture conditions

**DOI:** 10.1186/s12870-023-04461-y

**Published:** 2023-09-28

**Authors:** Wenxue Duan, Haiyan Zhang, Qingmei Wang, Beitao Xie, Liming Zhang

**Affiliations:** 1 Crop Research Institute, Shandong Academy of Agricultural Sciences, No.202 Gongyebei Road, Jinan, 250100 Shandong P. R. China; 2 Scientific Observation and Experimental Station of Tuber and Root Crops in Huang-Huai-Hai Region, Ministry of Agriculture and Rural Affairs, Jinan, 250100 China; 3 Shandong Engineering Laboratory for Characteristic Crops, Jinan, 250100 China; 4 Shandong Academy of Agricultural Sciences, No.202 Gongyebei Road, Jinan, 250100 Shandong P. R. China; 5 College of Life Sciences, Shandong Normal University, Jinan, 250014 China

## Abstract

**Background:**

Due to unreasonable nitrogen (N) application and water supply, sweet potato vines tend to grow excessively. Early development of storage roots is conducive to inhibiting vine overgrowth. Hence, we investigated how N and soil moisture affect early root growth and development.

**Results:**

A pot experiment was conducted using the sweet potato cultivars Jishu26 (J26, N-susceptible) and Xushu32 (X32, N-tolerant). Two N application rates of 50 (N1) and 150 mg kg^− 1^ (N2) and two water regimes, drought stress (DS) (W1) and normal moisture (W2), were applied to each cultivar. For J26, the lowest expansion root weight was observed in the N2W2 treatment, while for X32, the N1W2 and N2W2 treatments resulted in higher root weights compared to other treatments. The interaction between N rates and water regimes significantly affected root surface area and volume in J26. Root cross-sections revealed that N2W2 increased the percentage of root area covered by xylem vessels and decreased the amount of secondary xylem vessels (SXV) in J26. However, in X32, it increased the number of SXV. A high N rate reduced the ^13^ C distribution ratio in J26 expansion roots, but had no significant effect on X32. In J26, N2W2 inhibited starch synthesis in roots by downregulating the expression of *AGPa, AGPb, GBSS I*, and *SBE I*.

**Conclusion:**

The observed effects were more pronounced in J26. For X32, relatively high N and moisture levels did not significantly impact storage root development. Therefore, special attention should be paid to N supply and soil moisture for N-susceptible cultivars during the early growth stage.

**Supplementary Information:**

The online version contains supplementary material available at 10.1186/s12870-023-04461-y.

## Background

Currently, China leads the world in both the planting area and total sweet potato production [1]. The increasing demand for sweet potatoes in recent years can be attributed to their adaptability and nutritional value. Nitrogen (N) is an important nutrient element affecting sweet potato growth [2]. Previous studies have shown that a low N supply can stimulate root growth and differentiation in sweet potatoes, while high rates of N application can lead to root lignification and hinder storage root formation [3, 4]. Villordon et al. [3] proposed that an appropriate N application during the early growth stage enhances lateral roots and the number of adventitious roots. Reducing the base N supply can improve root volume and facilitate effective tuber formation during ridge closure [5]. Wang et al. [6] discovered that an optimal N supply promotes storage root development by promoting procambial cell differentiation and increasing the number of parenchyma cells in young roots. Different sweet potato cultivars exhibit varying N tolerance, with N-tolerant ones yielding more storage roots in response to elevated N [7]. Hence, N fertilizer management should account for cultivar-specific factors. Nonetheless, the impact of N on root differentiation and storage root development in cultivars with distinct N tolerances in sweet potatoes has been scarcely documented.

In most regions, sweet potatoes are predominantly rain-fed crops. Northern China, characterized by hilly terrain and infertile, drought-prone soil, extensively cultivates sweet potatoes. Spring rainfall in these areas is relatively low but coincides with sweet potato seedling planting. The critical period for water availability in sweet potatoes is during early growth, which significantly influences root system architecture establishment [8]. Early drought stress (DS) in sweet potatoes has notable effects on root growth, reducing the length of adventitious roots and the amount of lateral roots [9]. Villordon and colleagues [10] demonstrated that DS can induce cambium lignification around the primary xylem and secondary phloem in roots, while inhibiting storage root development. Wang et al. [11] reported that DS during the root establishment stage reduced the average root diameter and volume and hindered storage root development. Although the impact of DS on root morphological structure and growth indices has been studied, there is limited information regarding the effects of soil moisture on ^13^ C transfer allocation, root morphogenesis, and starch synthesis in storage roots during the early growth stage.

N supply and soil moisture are not only primary limiting factors but also interact closely with each other. Providing N and water in a balanced and sensible manner holds significant importance for enhancing crop growth and increasing yields [12]. Zhu et al. [13] demonstrated that ensuring an adequate supply of N and water effectively boosts dry matter accumulation (DMA) and enhances sweet potato root yields. Under DS conditions, a reasonable N supply could improve the photosynthetic rate, water use efficiency and activities of antioxidant enzymes in sweet potato leaves [14]. Previous research has primarily examined the alleviating effects of N application during DS at different growth stages. In actual sweet potato cultivation, due to irregular N application and uneven rainfall distribution, vine overgrowth is common, even in hilly regions. This can lead to yield reduction and the excessive use of plant growth retardants [7]. As a representative root tuber crop, the differentiation and development of storage roots significantly impact the final root yield. The early formation of storage roots is conducive to the inhibition of sweet potato overgrowth [15, 16]. Nevertheless, the regulatory mechanisms governing the impact of N and water on storage root development, particularly with regard to root differentiation and the formation of storage roots during the early growth phase, have seldom been explored. Furthermore, water can influence N absorption and utilization by affecting root growth and N transport within the soil. N utilization characteristics differ among various cultivars, and the cultivar factor should be taken into account. Thus, in this study, Jishu26 (J26, N-susceptible) and Xushu32 (X32, N-tolerant) were selected for pot experiments involving different N rates (50 and 150 mg kg^− 1^) and water regimes (DS and normal moisture). The aim was to examine disparities in their root growth characteristics, xylem development, ^13^ C distribution, starch accumulation, and transcript levels of genes regulating starch synthesis during the early growth stage. The results offer insights into the regulatory mechanisms underlying root differentiation and expansion responses to N and water and serve as a foundation for devising appropriate N and water management strategies tailored to sweet potato cultivars with varying N tolerances.

## Results

### FW of the shoots and roots

For both cultivars, W1 significantly decreased the FW of the shoots compared to W2 on all sampling days under the same level of N (Table 1). This direction also applied to the FW of the roots at 15 DAP and at 45 DAP for both cultivars and at 25 DAP for J26, respectively. At 25 DAP, W1 markedly reduced the FW of the thickening pigment roots compared to W2 under N1 for J26 and under N1 and N2 for X32. However, for J26, under N2, W2 had a significant decrease in the FW of these roots. Similar observations were noted at 45 DAP for the FW of the developing storage root.

**Table 1:** Fresh weight of shoots and roots during different growth stages under different treatments

Cultivar	Treatment	15 DAP			25 DAP				45 DAP		
		Fresh weight of shoot (g)	Fresh weight of root (g)		Fresh weight of shoot (g)	Fresh weight of root (g)	Fresh weight of thickening pigment root (g)		Fresh weight of shoot (g)	Fresh weight of root (g)	Fresh weight of developing storage root (g)
J26	N_1_W_1_	5.31 b	3.27 b		13.63 c	2.97 d	0.50 b		40.26 c	10.02 c	8.47 b
	N_1_W_2_	6.53 a	5.13 a		19.49 b	5.35 b	1.02 a		52.43 b	11.47 b	17.82 a
	N_2_W_1_	4.27 c	2.94 b		13.17 c	3.94 c	0.35 c		35.66 d	8.57 d	4.90 c
	N_2_W_2_	6.13 a	5.00 a		22.80 a	7.45 a	0.22 d		58.31 a	13.61 a	3.58 d
	N×W	NS	*		*	**	**		**	**	**
X32	N_1_W_1_	3.04 c	1.47 b		8.09 c	2.83 b	0.54 c		20.17 b	4.11 b	8.38 c
	N_1_W_2_	5.84 a	2.87 a		11.42 b	2.92 b	1.06 a		29.33 a	5.85 a	18.33 a
	N_2_W_1_	3.00 c	1.88 b		8.74 c	3.01 b	0.82 b		17.85 b	4.06 b	13.77 b
	N_2_W_2_	4.51 b	2.61 a		14.08 a	4.35 a	1.09 a		31.00 a	6.93 a	16.98 a
	N×W	NS	NS		*	**	*		NS	*	**

The values in the same column for each cultivar with different letters differ significantly as determined by Duncan’s multiple range test (P < 0.05)

The impact of N levels on the FW of the shoots and roots increased noticeably under W2 for J26 at 25 and 45 DAP, and for X32 under W2 at 25 DAP (Table 1). At 25 DAP, increasing the N rate significantly decreased the FW of thickening pigment roots under W1 and W2 for J26. However, for X32, a high N rate increased the FW of thickening pigment roots under W1 and exhibited no significant effect under W2. A similar trend was detected for the FW of developing storage roots at 45 DAP. The interaction between N rate and water regime had a significant impact on both parameters for both cultivars.

### Root length (RL), surface area (SA) and volume

Under the same N conditions, W2 remarkably induced the RL, SA and volume compared to W1 for both cultivars on all sampling days (Table 2). At 15 DAP, the effect of N rate on RL and SA was primarily seen in X32 under W1. For both cultivars, increasing the N rate significantly increased root volume under W1.

**Table 2:** The root length, root surface area, root volume, and its distribution ratio during different growth stages under different treatments

Cultivar	Treatment	Root length	Root surface area	Root volume	Distribution ratio of different diameters for root volume (%)			
		(cm)	(cm^2^)	(cm^3^)	0–2 mm	2–5 mm	5–20 mm	20–50 mm
15 DAP								
J26	N_1_W_1_	820.61 b	111.79 b	1.26 c	100			
	N_1_W_2_	1730.56 a	255.48 a	3.32 a	100			
	N_2_W_1_	950.96 b	131.72 b	1.63 b	100			
	N_2_W_2_	1809.50 a	262.28 a	3.44 a	100			
	N×W	NS	NS	NS				
X32	N_1_W_1_	476.71 d	69.85 c	0.89 c	100			
	N_1_W_2_	1491.19 a	195.71a	2.31 a	100			
	N_2_W_1_	1033.28 c	147.11 b	1.91 b	100			
	N_2_W_2_	1358.15 b	177.19 a	2.32 a	100			
	N×W	**	**	**				
25 DAP								
J26	N_1_W_1_	1609.37 d	258.65 d	4.16 d	85.33 c	14.67 a		
	N_1_W_2_	4283.82 b	679.10 b	10.58 a	86.21 c	11.11 b	2.67 a	
	N_2_W_1_	2659.74 c	416.02 c	5.98 c	91.58 b	8.42 c		
	N_2_W_2_	5035.14 a	752.81 a	9.76 b	97.40 a	2.60 d		
	N×W	NS	*	**	*	NS	**	
X32	N_1_W_1_	2073.22 b	268.90 c	3.48 d	87.29 a	12.71 b		
	N_1_W_2_	3329.41 a	429.54 b	6.42 b	73.12 c	15.40 a	11.47 a	
	N_2_W_1_	1852.48 b	281.38 c	4.61 c	82.81 b	17.19 a		
	N_2_W_2_	3150.55 a	480.28 a	7.60 a	82.36 b	7.95 c	9.69 b	
	N×W	NS	NS	NS	**	**	**	
45 DAP								
J26	N_1_W_1_	4114.05 c	655.94 c	17.28 b	43.53 c	9.37 b	47.10 b	
	N_1_W_2_	5852.32 b	897.27 b	28.40 a	33.80 d	5.69 c	60.51 a	
	N_2_W_1_	3503.87 d	593.11 c	13.66 c	58.96 b	14.57 a	26.48 c	
	N_2_W_2_	6803.43 a	1044.72 a	17.03 b	74.75 a	16.54 a	8.71 d	
	N×W	**	*	**	**	**	**	
X32	N_1_W_1_	1631.21 d	262.59 d	10.48 c	25.16 a	6.55 a	68.30 d	
	N_1_W_2_	2545.51 b	414.97 b	26.07 a	15.24 c	5.42 b	79.34 b	
	N_2_W_1_	2072.40 c	319.22 c	21.56 b	13.68 c	1.62 c	84.70 a	
	N_2_W_2_	3226.65 a	506.92 a	25.62 a	21.54 b	2.16 c	76.08 c	0.22 a
	N×W	NS	NS	**	**	**	**	**

The values in the same column for each cultivar with different letters differ significantly as determined by Duncan’s multiple range test (P < 0.05)

At 25 DAP, the impact of N rates on root SA was evident under W1 and W2 for J26 and under W2 for X32 (Table 2). An increase in N rates significantly decreased root volume under W2 for J26 but increased it under W1 and W2 for X32. N rates and soil moisture exhibited significant effects on root SA and volume for J26 alone. For each cultivar, N1W2 had the maximum distribution ratio (DR) of root volume within the 5–20 mm diameter range. Interestingly, for J26, N2W2 showed no roots within the 5–20 mm diameter range, while X32 displayed roots in this range under this treatment.

At 45 DAP, the impact of N rates on RL and SA increased under W2 for J26 and under W1 and W2 for X32 (Table 2). Increasing the N rate significantly decreased the root volume for J26 but increased it under W1 for X32. N and water exhibited significant interaction effects on these parameters for J26, but only on root volume for X32. For J26, N1W2 had the maximum DR of root volume within the 5–20 mm diameter range. Conversely, for X32, N2W1 showed the maximum DR of root volume within the 5–20 mm diameter range, followed by N1W2 and N2W2.

^**13**^** C DR in organs**.

**Fig. 1:**
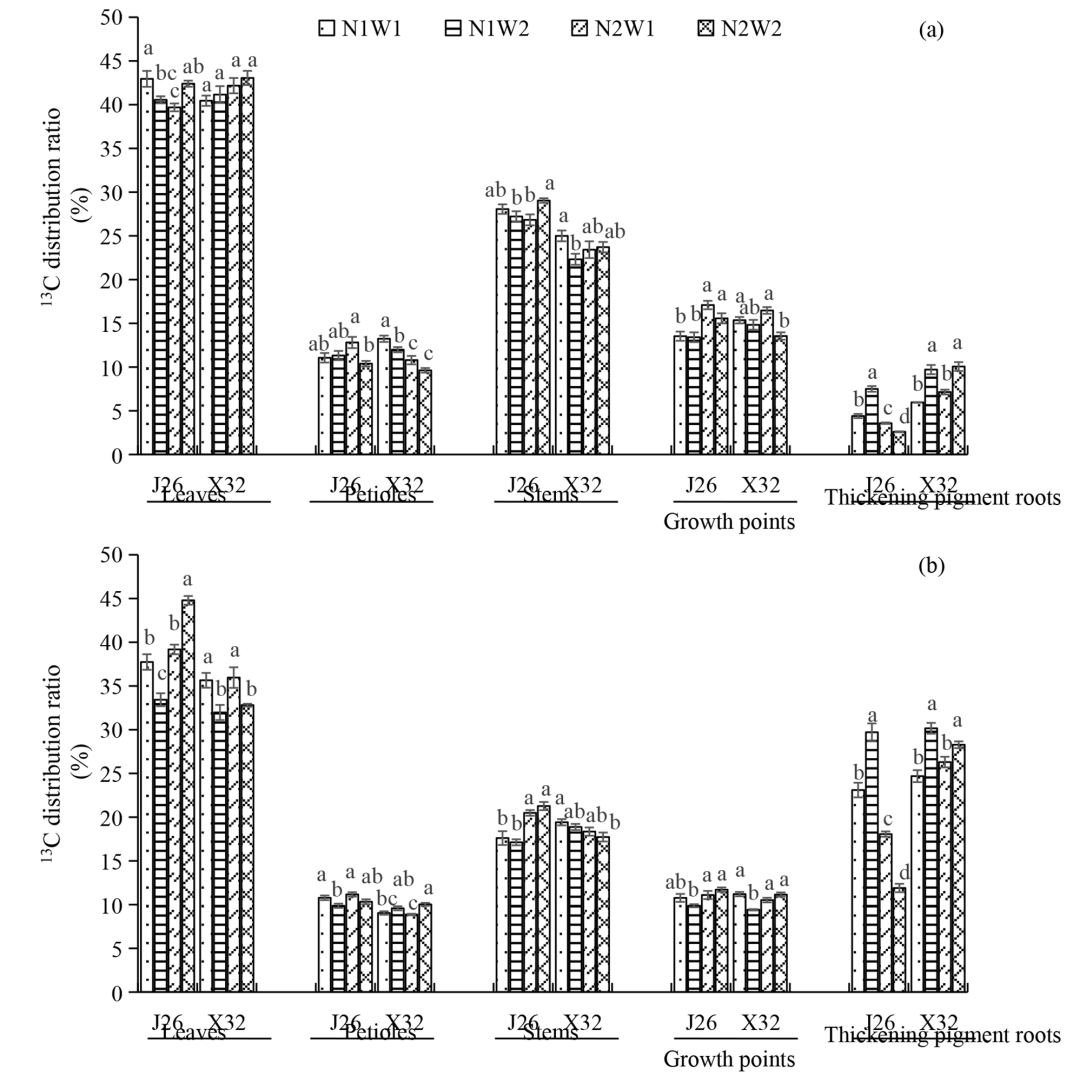
^13^ C distribution ratio in different organs at 25 DAP (a) and 45 DAP (b) under different treatments. The means of the same organ in each cultivar indicated by different letters are statistically significant (P < 0.05) as determined by Duncan’s multiple range test. Error bars represent standard errors of the means

At 25 DAP, compared to W1, W2 led to a significant increase in the ^13^ C DRs in the leaves and stems under N2 for J26, while decreasing these ratios in the petioles and stems under N1 for X32 (Fig. 1a). In the thickening pigment roots, for J26, W2 markedly elevated the ^13^ C DR under N1, but decreased it under N2. For X32, regardless of N levels, W2 significantly increased these values. Increasing N rates had a significant effect on increasing the ^13^ C DR in growth points for J26. However, for X32, the N rate did not significantly affect the ^13^ C DR in the leaf, stem, and thickening pigment root. The effect of N-water interaction significantly influenced the ^13^ C DR in the leaf, petioles, stem, and thickening pigment root for J26, while it was observed only in the growth points for X32.

At 45 DAP, compared to W1, W2 remarkably elevated the ^13^ C DR in the leaves and petioles for J26 and X32, respectively, under N2 (Fig. 1b). For J26, N2 markedly induced the ^13^ C DR in the leaves and growth points under W2 and in the stems under both W1 and W2. The changing trends in the ^13^ C DR in the developing storage roots were similar to those in the thickening pigment root at 25 DAP. The effect of N-water interaction was significant for the ^13^ C DR in the developing storage root of the two cultivars.

### Root xylem development at 25 DAP

**Fig. 2:**
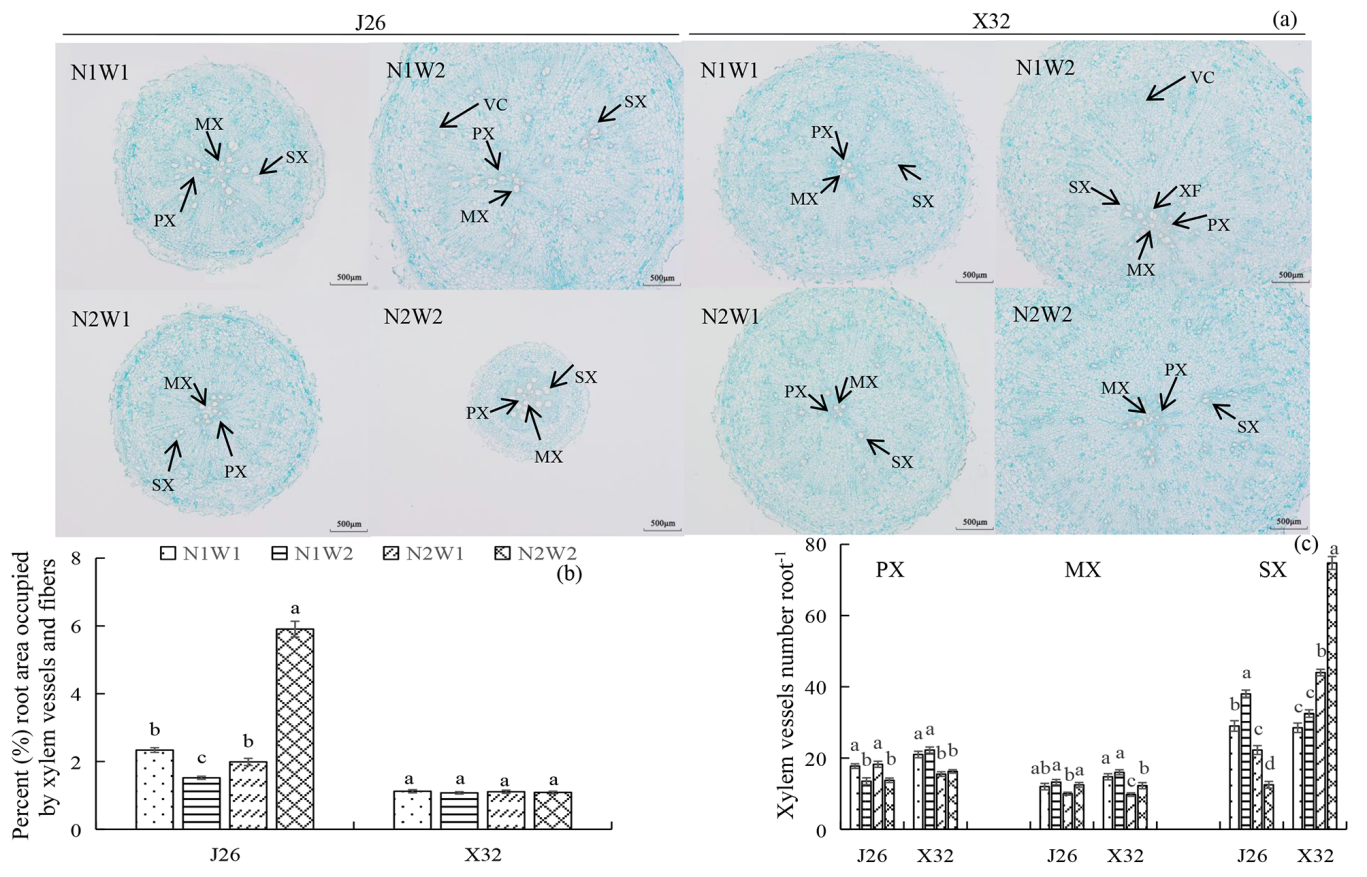
Root anatomy and xylem development under different treatments sampled at 25 DAP. (a) Depiction of cross-sections of adventitious roots obtained at 25 DAP. (b) Percentage (%) of root area occupied by xylem vessels, including protoxylem, metaxylem and secondary xylem, and xylem fibers at 25 DAP. (c) Number of xylem vessels number per root, encompassing protoxylem, metaxylem and secondary xylem. The means of each cultivar indicate by different letters are statistically significant (P < 0.05) as determined by Duncan’s multiple range test. Error bars represent standard errors of the means. PX, protoxylem; MX, metaxylem; SX, secondary xylem; XF, xylem fibers; VC, vascular cambium. Scale bar = 500 μm

Root anatomy and xylem development under different treatments for each cultivar are illustrated in Fig. 2a. As compared to W1, W2 significantly reduced the percentage of root area occupied by xylem vessels under N1, but increased this parameter under N2 for J26 (Fig. 2b). For J26, increasing the N rate significantly increased the percentage of root area occupied by xylem vessels under W2. For X32, no obvious differences were found among the different treatments. The effect of N-water interaction occurred only for J26.

Compared to W1, W2 significantly decreased the number of protoxylem (PX) vessels for J26, but had no obvious effect on these vessels for X32 (Fig. 2c). For both cultivars, W2 significantly increased the number of metaxylem (MX) vessels under N2. Increasing the N rate significantly decreased the number of PX and MX vessels for X32. Compared to W1, W2 significantly increased the number of secondary xylem (SX) vessels under N1 for J26 and under N2 for X32. However, W2 significantly decreased those under N2 for J26. Increasing the N rate significantly decreased the number of SX vessels for J26, while the opposite results were observed for X32. The effect of N-water interaction on the number of SX vessels occurred in both cultivars.

### Starch content and enzyme activities related to starch synthesis at 45 DAP

**Fig. 3:**
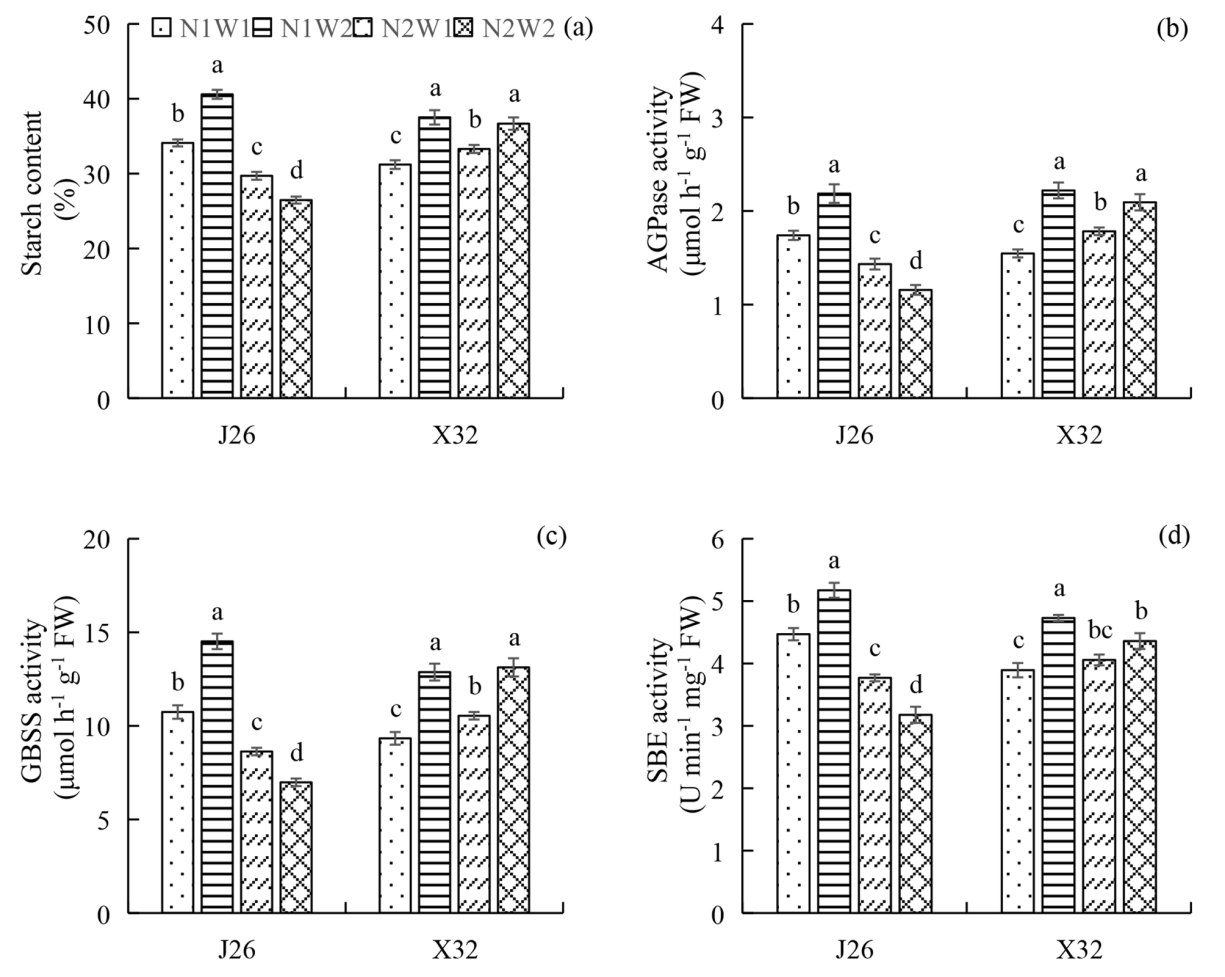
The starch content (a), activities of AGPase (b), GBSS (c) and SBE (d) at 45 DAP under different treatments. The means of each cultivar indicated by different letters are statistically significant (P < 0.05) as determined by Duncan’s multiple range test. Error bars represent standard errors of the means

Compared to W1, W2 significantly increased the starch content under N1 for J26 and under N1 and N2 for X32 (Fig. 3a). However, W2 significantly decreased the starch content under N2 for J26. Increasing the N rate significantly decreased the starch content of J26. However, for X32, the starch content was significantly increased under W1, but showed no significant effect under W2. The effect of N-water interaction on starch content occurred only for J26. Similar trends were observed for the activities of AGPase and GBSS for each cultivar (Fig. 3b and c). Compared to W1, W2 significantly increased the SBE activity under N1 for both cultivars, while decreasing it under N2 for J26 (Fig. 3d). Soil moisture exhibited no significant effect on these under N2 for X32. Increasing the N rate significantly decreased the SBE activity under W1 and W2 for J26 and under W2 for X32. The N rate exhibited no significant effect on these activities under W1 for X32. The effects of N-water interaction were significant for the AGPase and SBE activities of both cultivars and for the GBSS activity of J26 alone.

### Expression of genes related to starch synthesis at 45 DAP

**Fig. 4:**
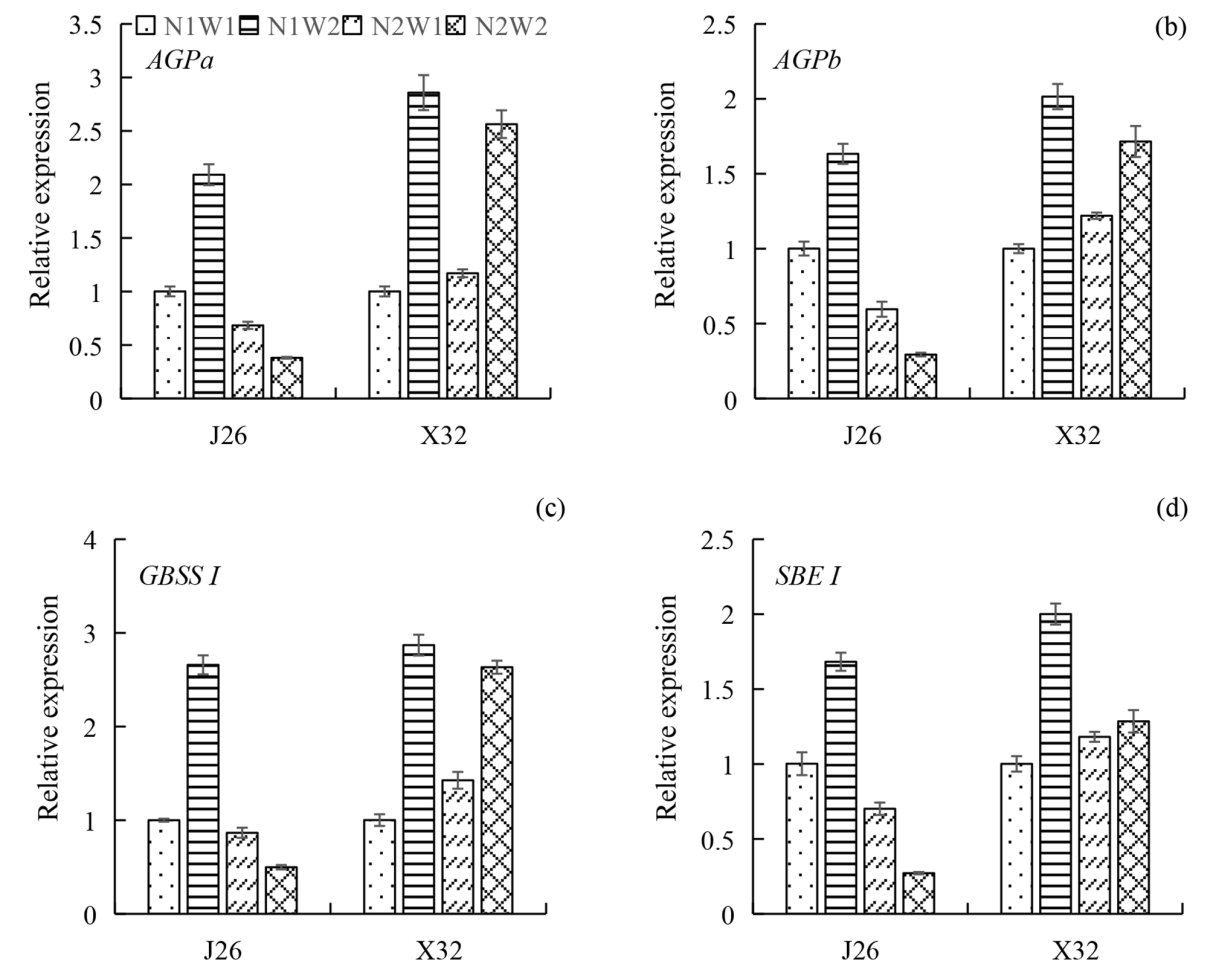
The relative expression of genes *AGPa* (a)、*AGPb* (b)、*GBSS I* (c) and *SBE I* (d) under different treatments. The means of each cultivar indicated by different letters are statistically significant (P < 0.05) as determined by Duncan’s multiple range test. Error bars represent standard errors of the means

Compared to W1, W2 upregulated the expression of *AGPa* by 2.09-fold under N1 for J26 and by 2.86- and 2.19-fold under N1 and N2 for X32, respectively (Fig. 4a). However, W2 significantly downregulated the expression of this gene in J26 under N2. Increasing the N rate reduced the expression of *AGPa* in J26, but had no obvious effect in X32. Similar trends were observed for the expression of *AGPb* (Fig. 4b).

Compared to W1, W2 increased the expression of *GBSS I* by 2.66-fold under N1 for J26 and by 2.87-fold under N1 and 1.85-fold under N2 for X32 (Fig. 4c). N2W2 showed the lowest expression of *GBSS I* for J26, while no significant difference was observed between N1W2 and N2W2 for X32. For J26, the changing trends in the expression of *SBE I* were similar to those for the *GBSS I* gene (Fig. 4d). A high N rate downregulated the expression of *SBE I* under W2 in X32.

## Discussion

RL, SA and volume are key morphological indicators of root development. Enhanced root SA and volume contribute to increased nutrient absorption capacity in roots, positively impacting sweet potato yields [5, 17]. N fertilizer plays a significant role in the growth of sweet potato roots. Villordon and colleagues [18] demonstrated that an application of 50 kg N ha^− 1^ increased lateral RL and SA, resulting in higher final yields. Chen and co-workers [17] showed that the use of 90 kg N ha^− 1^ reduced DMA in storage roots along with their SA, length, and volume during the early stages of root formation. Herein, the effect of N supply on root parameters varied between different cultivars throughout the root growth process (Table 2). At 15 DAP, the N rate showed no significant effect on RL and SA for J26. In contrast, a high N rate increased these parameters under DS for X32. At 25 DAP, increasing the N rate increased the RL and SA for J26. However, for X32, a high N rate exhibited no significant effect on RL but increased root volume. At 45 DAP, the high N rate reduced the root volume for J26, and the positive effects on X32 disappeared. These results suggest that a high N rate, combined with normal soil moisture, leads to greater RL and SA in the N-susceptible cultivar, potentially resulting in increased N absorption and promoting shoot growth (Table 1). However, higher RL and SA may not be associated with higher FW of the expansion roots (Table 1). In sweet potatoes, early exposure to DS exhibited a more pronounced impact on decreasing RL, SA and volume [14]. Wang et al. [11] found that DS at 10 DAP significantly reduced the root volume, followed by 20 and 30 DAP. In the present study, for both cultivars, DS from 10 to 15 DAP decreased RL, SA and volume, and the inhibitory effects on root expansion could still be observed even at 45 DAP (Table 2). Villordon et al. [10] reported that even under optimal substrate moisture conditions, variations in N rates and local nutrient availability significantly influenced root architecture attributes during storage root development. At 25 DAP, N and water exhibited significant interactive effects on root SA and volume for J26. However, there were no significant interactive effects on these parameters for X32, indicating that N and water more strongly regulated root growth in J26. Therefore, greater attention should be paid to N and water supply during the early growth stages of the N-susceptible cultivar. In the present study, we also examined root volume distribution at varying diameters. At 25 DAP, for J26, increasing the N rate or soil moisture decreased the DR of root volume for roots (2–5 mm in diameter). Conversely, for X32, increasing the N rate increased the DR of root volume for roots (2–5 mm in diameter) under W1. Relatively high N and soil moisture inhibited root expansion of J26, while increasing the N rate under DS promoted root expansion in X32.

Previous research has demonstrated a close relationship between the movement and distribution of photosynthetic products from leaves to root tubers and various factors, including genotype characteristics, soil N nutrition, and soil moisture [7, 19]. High-yielding sweet potato cultivars tend to become hubs for photosynthate supply in their storage roots earlier than low-yielding cultivars [15]. Taranet et al. [20] showed that excessive N supply can restrict the utilization of photoassimilates during the period of storage root bulking, ultimately reducing storage root yield. Duan et al. [7] reported that an abundant supply of N reduced the allocation of ^13^ C to storage roots due to an increase in allocation to aboveground parts, particularly stems, leaves, and branch growth points. Root and tuber crops are more susceptible to yield reductions when drought occurs during the formation of storage roots [21]. Zhang and co-workers [22] discovered that DS during the initiation of storage root growth led to a reduction in the transfer of assimilation products to storage roots, leading to a significant reduction in yields. DS in the medium term decreased the rate of ^13^ C allocation to storage roots, while moderate irrigation facilitated the transport of assimilation products to storage roots [23]. In the present study, the ^13^ C DR in expansion roots increased during the growth process, with N and soil moisture affecting it differently across various cultivars (Fig. 1). At 25 DAP, increasing the N rate decreased the ^13^ C DR in thickened roots for J26, but had no significant effect on those for X32. For J26, DS decreased the ^13^ C DR in thickened roots under N1, but increased it under N2. However, for X32, DS decreased these values regardless of the N level. Similar results were observed for the ^13^ C DR in the developing storage roots at 45 DAP. Analysis of the ^13^ C DR in aboveground organs revealed that N application to J26 mainly elevated the ^13^ C DR in growth points at 25 DAP and in both stems and growth points at 45 DAP (Fig. 1), indicating a relatively robust metabolism of growth points in this N-sensitive cultivar. Therefore, a high N rate promoted shoot elongation. Zhu et al. [13] reported that N application reduced DMA in storage roots under low soil moisture conditions, but increased it under high soil moisture conditions, highlighting the importance of sufficient nutrients and water for achieving high sweet potato yields. Our results showed that N and water exhibited a significant interaction effect on the ^13^ C DR in J26 expanded roots at both 25 and 45 DAP, whereas this interaction effect was only present for X32 at 45 DAP. This suggests that N rates and soil moisture significantly affected carbon supply in the expanded roots of J26 during the early growth stage. For an N-susceptible cultivar, a high N supply combined with normal soil moisture did not facilitate the transport of assimilation products from the leaves to expanded roots. Instead, these conditions promoted the retention of more assimilation products in stems and growth points, which are other metabolic centers, resulting in vigorous vegetative growth and inhibited storage root formation.

The process of lignification in the stele during the early stages of root formation has a direct influence on storage root development [24]. In sweet potato roots, the stele tissue undergoes lignification, preventing it from expanding due to the absence of anomalous cambia and vascular development [18]. This connection between lignification and xylem development has been observed in various plant systems [25, 26]. Notably, a significant metabolic shift towards the deposition of secondary cell walls occurs during the maturation of interfascicular fiber cells, xylary fiber cells, and xylem vessels [27]. An excessive supply of N may hinder cell division in the stele, thereby promoting the formation of non-storage roots. In our current study, an increase in N levels led to a greater proportion of the root area covered by xylem vessels under normal moisture conditions for J26 (Fig. 2b), while the interaction effect of N-water on these showed no significant differences for X32, indicating that a high N rate combined with normal soil moisture resulted in a higher degree of lignification in J26’s roots. A well-developed secondary cambium with strong activity plays an essential role in promoting the formation and expansion of storage roots [24]. Wang et al. [6] demonstrated that a low N rate increased the number of secondary vascular bundles and promoted their distribution in the stellar tissue. This treatment led to the formation of more SX and the surrounding parenchymal tissue, ultimately promoting the activity of secondary cambium and supporting storage root development. In the present study, N rates and soil moisture exhibited different regulatory effects on SX development for each cultivar (Fig. 2c). For J26, increasing the N rate reduced the number of SX vessels, especially under normal moisture conditions. Conversely, for X32, a high N rate resulted in an increase in this parameter. It is important to note that higher N and soil moisture were not conducive to the division of secondary cambium for an N-susceptible cultivar.

The growth of sweet potato storage roots is intricately linked to the capacity of the developing storage root to act as a sink for nutrients [28]. Carbon-metabolizing enzymes play a crucial role in regulating the turgor pressure gradient between source and sink organs in plants by controlling the rates of sucrose and starch synthesis. Pinheiro and colleagues [29] showed that sucrose is converted into starch within storage roots under the influence of AGPase, suggesting that the increased activity of AGPase is beneficial for improving the sink strength. Elevated expression levels of genes responsible for starch metabolism indicate increased starch synthesis and promote the formation of storage roots [30]. Ravi and colleagues [31] found that *SBE*, *GBSS*, and *AGPase* were upregulated during the development of storage roots. *AGPase* was found to be upregulated in enlarged storage roots, while *GBSS I* expression increased during the rapid bulking phase of storage root growth [32, 33]. Improving the enzymatic activity and gene expression of *SBE* led to a higher starch yield [34]. Du and co-workers [16] reported that AGPase acts as the rate-limiting enzyme in starch synthesis within storage roots under various N management strategies. A reduced supply of N could upregulate the *AGPase* gene and enhance AGPase enzymatic activity, ultimately promoting starch accumulation in the storage roots. Kim and colleagues [35] observed that DS reduced the expression of the *AGPase* gene in the thickened roots of sweet potatoes. According to Li et al. [23], appropriate irrigation increased AGPase activity and promoted starch synthesis in storage roots. In the present study, N and water affected starch synthesis in the expansion roots for both cultivars (Fig. 3). Increasing the N rate decreased the expression of *AGPa, AGPb, GBSS I*, and *SBE I* under normal moisture conditions for J26, but it had no obvious effects on *AGPa*, *AGPb* and *GBSS I* for X32 (Fig. 4). DS decreased the expression of these four genes under low N conditions in J26 and decreased the expression of *AGPa, AGPb* and *GBSS I* in X32 regardless of N levels. The alterations in the levels of AGPase, GBSS and SBE were mainly consistent with the changes in gene expression. For both cultivars, the effects of N-water interaction were significant for AGPase and SBE activities. N rates and soil moisture regulated sink strength by affecting the activities of the key enzymes responsible for starch synthesis, which are closely related to the turgor pressure gradient governing nutrient transport between the leaf and storage root [23, 29]. Consequently, this regulation affects the carbon flow to the roots, influencing the expansion of storage roots.

## Conclusion

The regulation of early root development had a more pronounced impact on the N-susceptible cultivar J26. In the case of J26, N2W2 promoted root elongation, caused root lignification, reduced starch accumulation and limited the assimilates transported to the roots, ultimately inhibiting the development of storage roots. However, for X32, relatively high levels of N and moisture did not seem to significantly affect storage root development. In fact, a high N rate promoted storage root development in this cultivar under DS. Thus, more attention should be paid to the supply of N and water, especially for N-susceptible cultivars during the early growth stage.

## Materials and methods

### Experimental design

A pot experiment was conducted under a rainproof shed at the Shandong Academy of Agricultural Sciences, China (36°7′ N, 118°2′ E) in 2021. The sweet potato cultivars chosen for this study were Jishu 26 (J26, N-susceptible) and Xushu32 (X32, N-tolerant), which had previously been confirmed in prior experiments [7, 36]. To conduct the study, plastic pots with dimensions of 40 cm in inner diameter and 35 cm in height were buried in the ground for planting sweet potato plants. Each pot contained 20 kg of soil. The pot soil consisted of 9.8 g kg^− 1^ organic matter and 59.2, 20.8 and 93.2 mg kg^− 1^ alkali-hydrolysable N, available phosphorus (P) and available potassium (K), respectively. In the experiment, two levels of nitrogen (N) were employed: 1.0 and 3.0 g of N were applied to each pot, corresponding to N rates of 50 (N1) and 150 (N2) mg kg^− 1^, respectively. Each pot accommodated a single sweet potato plant, and the relative soil water content (RSWC) was adjusted to 70% after planting. Two water regimes were set up 10 days after planting (DAP), the early DS with the RSWC adjusted to 55% for five days (T0) and the normal water condition with a RSWC of 70% during this period (T1). The remaining growth periods of the RSWC were consistent at 70%. The soil moisture content was determined using a HH_2_ soil moisture measurement device (Delta-T Devices LTD, Cambridge, UK). The amount of water required for supplemental irrigation was evaluated according to the mathematical formula developed by Ekren and co-workers (2012). The treatments received an identical amount of K and P fertilizers. Each pot was supplied with 1.5 g of P_2_O_5_ and 3.0 g of K_2_O as base fertilizers. These fertilizers consisted of potassium sulfate (50% K_2_O), calcium triple superphosphate (46% P_2_O_5_), and urea (46.4% N). The pots were randomly arranged in sets of three replicates, resulting in a total of 176 pots for each replicate.

### Sampling approaches

In each replicate of every treatment, three plants chosen at random were gathered for root scanning at 15 DAP. At 25 DAP, five randomly chosen plants were harvested for root scanning and microstructure preparation. Similarly, at 45 DAP, three randomly selected plants were harvested for root scanning. Additionally, another three randomly chosen plants were harvested, and their middle sections, comprising 1 cm segments of the developing storage roots, including the skin, were uniformly mixed to determine physiological indices and conduct gene expression analysis. This procedure was replicated three times for each time period.

### Variable measurements

#### Fresh weights (FW) of the roots and shoots

The fresh weights of the roots and shoots were recorded on all sampling days. Moreover, the FW of the thickening pigment root was measured at 25 DAP, and the FW of the developing storage root was measured at 45 DAP.

^**13**^** C labeling**.

^13^ C labeling of sweet potato plants was conducted at 25 and 45 DAP. A total of 3 plants were selected, and the 4th and 5th fully expanding leaves on the main stem, starting from the shoot apex, were enclosed in an airbag. These leaves were suspended within the airbag, which had a volume of 400 mL. ^13^CO_2_ gas (50 mL, 8%) was injected into each airbag, making up ~ 1% of the total gas volume. The ^13^CO_2_ gas (with 99% atom ^13^ C) was sourced from the Shanghai Engineering Research Center of Stable Isotope. After labeling for 1 h, the airbag was removed. Following a 48-h interval, the plants were uprooted randomly to harvest the expansion roots and aboveground organs. The leaves, petioles, stems, growth points (the section above the 5th fully expanded leaf from the shoot apex) and expansion roots were collected, dried in the oven and then ground into powders by using a Waring blender. The obtained powders were then evaluated using a stable-isotope-ratio-mass-spectrometer [15].

### Root anatomical structure

The less than 1 cm long pieces from the middle parts of the thickest roots were cut at 25 DAP and fixed in FAA fixation solution to make paraffin sections. Staining with safranin and fast green was conducted on each section and the anatomical structure was observed and photographed using an Olympus BX53 optical microscope, and the quantitative indices were assessed by Image-Pro Plus v6.0 image processing software.

### Root growth properties

Root specimens were subjected to scanning using a scanner (LA1600 + scanner, Canada) and subsequently evaluated using WinRHIZO software (Regent Instruments Inc., Quebec, Canada) to determine RL, volume, SA, and volume distribution across various diameter ranges [17].

### Starch contents and related enzyme activities during starch synthesis

The starch content was detected as described previously [37]. The enzyme extraction solution was prepared following Yang et al. [38]. The root samples were frozen using liquid N and subsequently pulverized. One-gram samples were then extracted using 10 mL of extraction buffer with a pH of 7.5, composed of 100 mM HEPES-NaOH, 8 mM MgCl2, 50 mM 2-mercaptoethanol, 2 mM EDTA, 12.5% (v/v) glycerol, and 5% (w/v) insoluble polyvinylpyrrolidone. Following extraction, the samples were incubated on ice and then subjected to centrifugation (10,000 g, 4 °C, 10 min). The obtained precipitate was resuspended in an extraction buffer and utilized to prepare granule-bound starch synthase (GBSS). The remaining homogenate was once again centrifuged (10,000 g, 4 °C, 10 min), and the resulting supernatant was harvested for the analysis of enzyme activities, including starch branching enzyme (SEB) and ADP glucose pyrophosphorylase (AGPase). AGPase activity was determined following the method described by Wang and colleagues [39], while the activities of SBE and GBSS were measured according to the procedure outlined by Jiang and colleagues [40].

### qRT–PCR assay

The expression levels *SBE I*, *GBSS I*, *AGPa*, and *AGPb* were evaluated utilizing a qRT–PCR analysis. Total RNA extraction was carried out with the corresponding kit (Tiangen Biotech, China) in accordance with the manufacturer’s guidelines. Subsequently, cDNA synthesis was conducted (Takara Bio, Japan). qRT–PCR was performed on a Bio–Rad CFX96 thermocycler (Bio–Rad, Hercules, CA, USA) using SYBR green fluorescent dye and. To standardize the gene expression data, they were normalized relative to the *IbActin* expression level. The results from the qRT–PCR analysis were analyzed using the 2^−ΔΔ^CT method [41]. Detailed primer information can be found in Supplementary Table S1.

### Statistical analysis

To test the treatment effects, ANOVA was performed for each cultivar in a two-factor randomized complete design, utilizing SPSS software (v17.0 for Windows, USA). Mean differences and interactions between N and water (N-water) for the treatments were examined using Duncan’s multiple range test, with significance set at P < 0.05.

Figure 1. ^13^ C distribution ratio in different organs at 25 DAP (a) and 45 DAP (b) under different treatments. The means of the same organ in each cultivar indicated by different letters are statistically significant (P < 0.05) as determined by Duncan’s multiple range test. Error bars represent standard errors of the means.

### Electronic supplementary material

Below is the link to the electronic supplementary material.

Supplementary Material 1

## Data Availability

The datasets utilized and/or analyzed in this study can be acquired from the corresponding author upon a reasonable request.
