# Supplementary material for: Regulation of root development in nitrogen-susceptible and nitrogen-tolerant sweet potato cultivars under different nitrogen and soil moisture conditions
Source: BMC Plant Biol. 2023 Sep 28;23:454. doi: 10.1186/s12870-023-04461-y (PMC10537907; doi:10.1186/s12870-023-04461-y)
Supplement: Supplementary file 1 — Supplementary Material 1 [file 12870_2023_4461_MOESM1_ESM.doc]

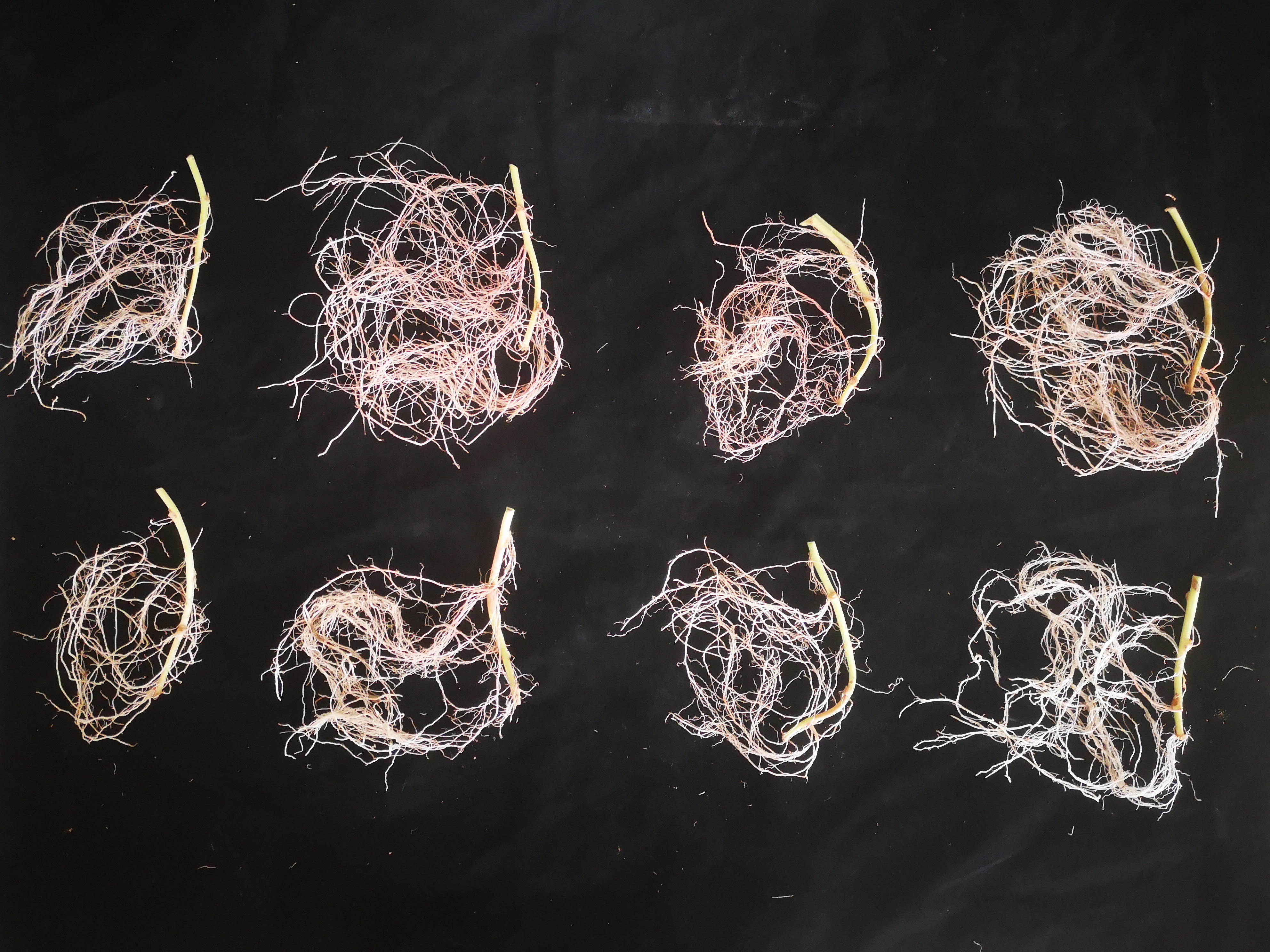


**N1W1**

**N1W2**

**N2W1**

**N2W2**

**X32**

**J26**

**15 DAP**


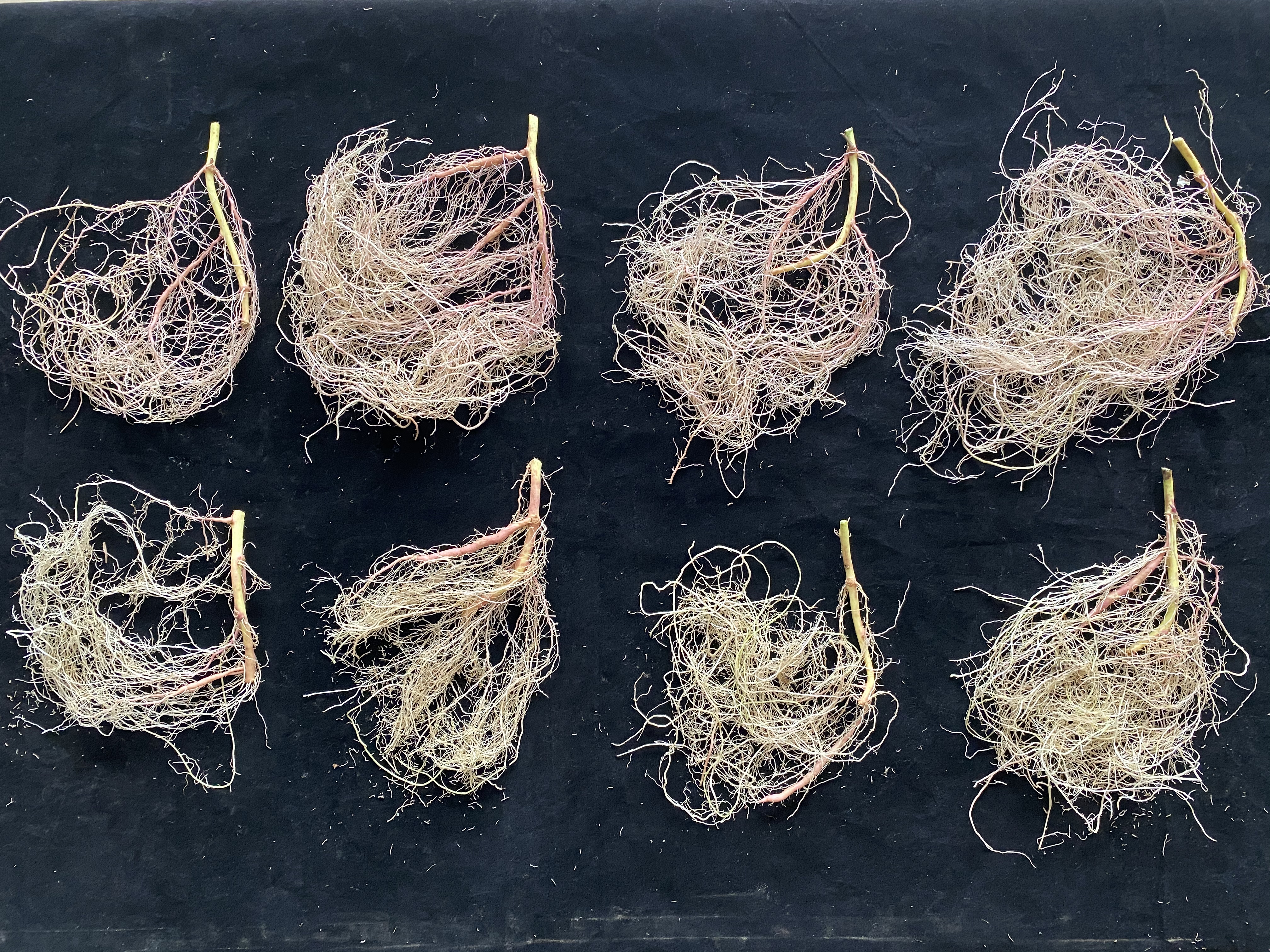


**N1W1**

**N1W2**

**N2W1**

**N2W2**

**X32**

**J26**

**25 DAP**


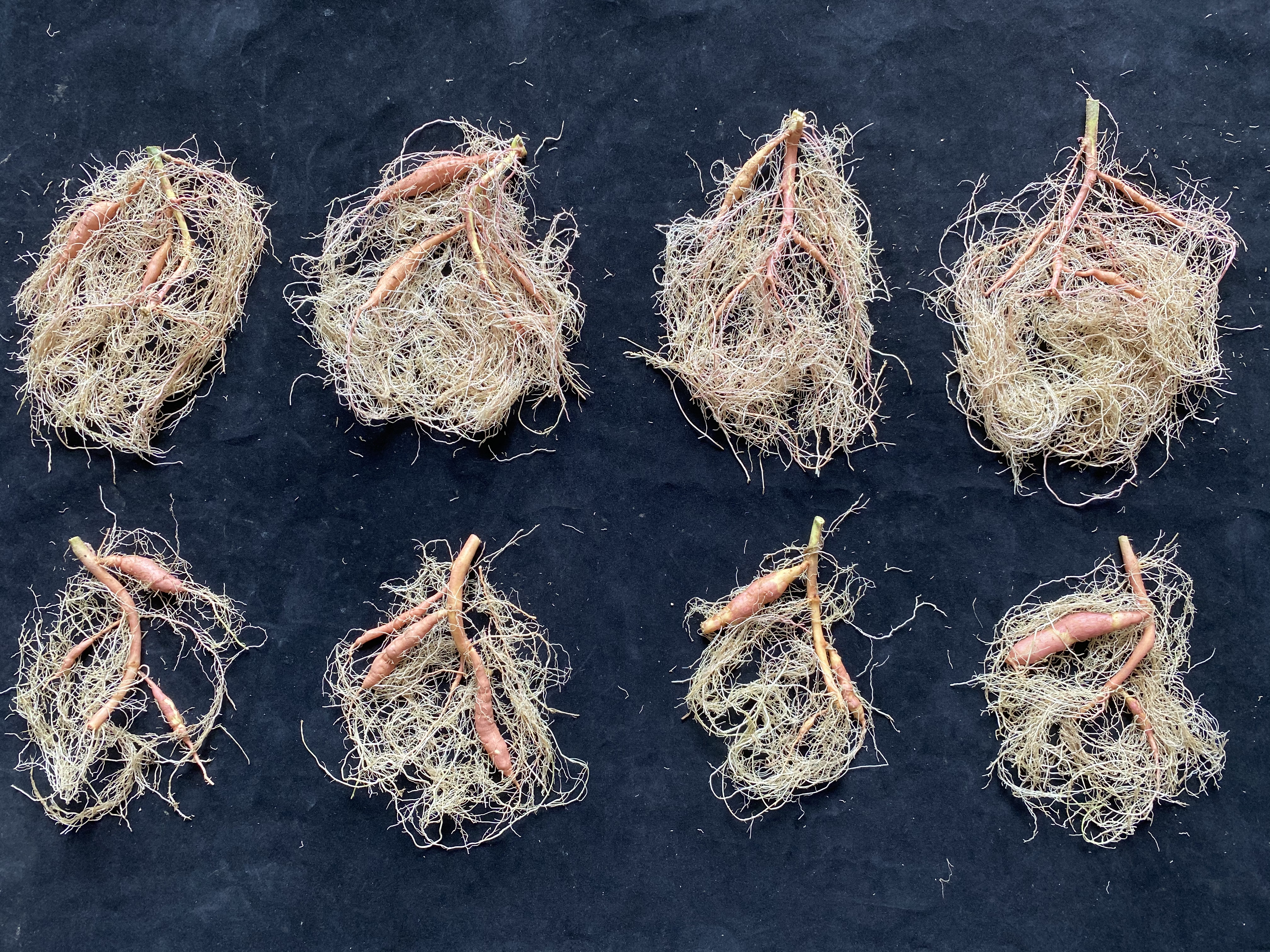


**N1W1**

**N1W2**

**N2W1**

**N2W2**

**X32**

**J26**

**45 DAP**

Figure S1. The storage root development during different growth stages under different treatments.

Table S1. Primers used for RT-PCR analysis.

| ***Primer*** | ***Forward primer (5’→3’)*** | ***Reverse primer (5’→3’)*** |
| --- | --- | --- |
| ***IbActin*** | CTGGTGTTATGGTTGGGATGG | GGGGTGCCTCGGTAAGAAG |
| ***IbAGPa*** | TCGACGGTGATGTTAGCAAG | AACAGCCTTTGGAGAAACGA |
| ***IbAGPb*** | GACAAGAACGTAAGGATTGGGA | CGAATGGTTGCTTTCTCCAT |
| ***IbGBSSI*** | CAGTTGGTTTGCCAGTTGAC | ACGTTGAACTTTGCCACTCC |
| ***IbSBEI*** | GGTTTACGGGTCTTGATGGA | AACAGCCTGCTATCCCACAC |
